# Driving the usage of tuberculosis diagnostic data through capacity building in low- and middle-income countries

**DOI:** 10.4102/ajlm.v9i2.1092

**Published:** 2020-11-18

**Authors:** Natasha Gous, Alaine U. Nyaruhirira, Bradford Cunningham, Chris Macek

**Affiliations:** 1 Global Health, SystemOne, LLC, Johannesburg, South Africa; 2 Management Sciences for Health, Pretoria, South Africa; 3 Strategic Initiatives, SystemOne, LLC, Johannesburg, South Africa; 4 Business Development, SystemOne, LLC, Northampton, Massachusetts, United States

**Keywords:** tuberculosis, GeneXpert, diagnostic data, monitoring and evaluation, data analysis, programmatic

## Abstract

**Background:**

Connectivity platforms collect a wealth of data from connected GeneXpert instruments, with the potential to provide valuable insights into the burden of disease and effectiveness of tuberculosis programmes. The challenge faced by many countries is a lack of training, analytical skills, and resources required to understand and translate this data into patient management and programme improvement.

**Objective:**

We describe a novel training programme, the tuberculosis Data Fellowship, designed to build capacity in low- and middle- income countries for tuberculosis data analytics.

**Methods:**

The programme consisted of classroom and remote training plus mentorship over a 12-month period. The focus was on skills development in Tableau software, followed by training in exploration, analysis, and interpretation of GeneXpert tuberculosis data across five key programme areas: patient services, programme monitoring, quality of testing, inventory management, and disease burden.

**Results:**

The programme was piloted in six countries (Bangladesh, Ethiopia, Ghana, Malawi, Mozambique) in July 2018 and Nigeria in September 2018; 20 participants completed the training. A number of key outputs have been achieved, such as improved instrument utilisation rates, decreased error rates, and improved instrument management.

**Conclusion:**

The training programme empowers local tuberculosis programme staff to discover and fix critical inefficiencies, provides high-level technical and operational support to the tuberculosis programme, and provides a platform for continued sharing of insights and best practices between countries. It supports the notion that connectivity can increase efficiencies and clinical benefits with better data for decision making, if coupled with commensurate capacity building in data analysis and interpretation.

## Introduction

Tuberculosis has been declared a global public health emergency. An estimated one-third of the world’s population is infected with tuberculosis; 10 million people developed tuberculosis disease in 2017 alone,^1^ a number that may be underestimated due to under-reporting and lack of reliable data. The widespread implementation of the Xpert® MTB/RIF assay (Cepheid, Sunnyvale, California, United States) for detection of *Mycobacterium tuberculosis* and rifampicin resistance as a first-line tuberculosis diagnostic, has been hailed as the most significant advancement in decades, becoming the first molecular assay to provide a tuberculosis and first-line drug resistance diagnosis in just 2 hours. Following widespread adoption of this technology, the World Health Organization’s Agenda for Action on Digital Health for the End Tuberculosis Strategy called for 100% of all sites using rapid tuberculosis diagnostic instruments to be connected by 2020,^2^ becoming the first to recognise the role of digital health in the fight against tuberculosis. Over the past 2 to 3 years, numerous countries have begun adopting connectivity platforms to help monitor and manage their GeneXpert fleet by collecting the vast amounts of rich clinical diagnostic and operational data produced by the instrument. Rarely before has such a rich data resource been both produced by a diagnostic instrument and been made available via connectivity platforms, at scale, for analysis. As yet, it remains largely untapped.^3^ If these data can be analysed, interpreted and translated into appropriate recommendations and actions, they have the potential to provide significant transformative impact on the management and effectiveness of infectious disease programmes worldwide.

GxAlert® (SystemOne, LLC, Northampton, Massachusetts, United States) is currently collecting data from GeneXpert platforms in 43 countries running Cepheid’s Xpert MTB/RIF assay. GxAlert is a connectivity platform that integrates directly with diagnostic instruments to collect and send a digital copy of test results and associated instrument metadata to an in-country or GxAlert server. From there, results can be sent and accessed via short message service and email alerts, Microsoft Excel (Microsoft Corp, Redmond, Washington, United States) reports and web dashboards. The type of data being collected includes not only the diagnostic result, but also information on when and where the test was run and by whom, demographic information about the patient (through an application called GxConnect), reagent lot numbers, probe data, cycle thresholds as well as instrument operational data such as instrument failures, inventory consumption and instrument downtime. From these data, critical insights can be gained or inferred about the tuberculosis programme and can help shed light on both clinical and operational return on investment. Data can also provide useful information on testing coverage, disease status and trends, circulating strains and drug resistance profiles, instrument utilisation rates, training needs, supply chain, inventory, and quality of the testing programme.^4^

But there is a challenge: even though countries now collect this type of data in large volumes, it is a new arena for them. Most high-disease burden countries lack the tools, resources and expertise required to analyse, understand and translate these data into improved programme and patient outcomes. A recent study by the Foundation for Innovative Diagnostics (FIND), found that despite large investments by donors to implement electronic data management systems, there is limited usage of the data to improve service delivery, mainly due to a lack of understanding and awareness of what data means.^5^ As a result, tuberculosis programmes are accumulating but not using the data being collected to drive decision making. This becomes apparent when one considers the various challenges still hindering tuberculosis programmes today, including gross under-utilisation of instruments,^3,6,7,8^ high unsuccessful test and error rates (loss of tests),^9,10^ cartridge stock-outs, instrument breakdowns, and lack of adequate module replacements and maintenance of instruments.^11^

There is a dire need to build capacity in health data analytical skills amongst staff within national tuberculosis programmes (NTPs) in order to bolster the usage of data. To address this need, we designed a novel training programme to develop the expertise and skills required for the analysis and understanding of connected diagnostic data.

## The Tuberculosis Data Fellowship programme

The TB Data Fellowship (TDF) programme was initiated in 2018 through a joint collaboration between SystemOne and Management Sciences for Health, with the support of the Tableau Foundation. Designed to build the foundation for sustainable in-country capacity, the programme aimed to enhance the understanding of tuberculosis data and its translation into actionable outputs. Achieving these goals required a new cadre of healthcare worker to be trained, one with the ability to understand and interpret the vast amounts of diagnostic and operational data being collected through connected diagnostic systems.

For the pilot programme, staff from the NTPs, national tuberculosis reference laboratories and ministries of health from several countries using the GxAlert connectivity platform were invited to apply. Countries invited included Bangladesh, Ethiopia, Ghana, Malawi, Mozambique and Nigeria. The selection criteria for the programme included a minimum of 2 years’ work experience in the field of tuberculosis, and more specifically the GeneXpert tuberculosis programme, and at least 6 months of work experience with GxAlert software. Participants also had to have experience working in Excel.

### Informatics infrastructure

**FIGURE 1:**
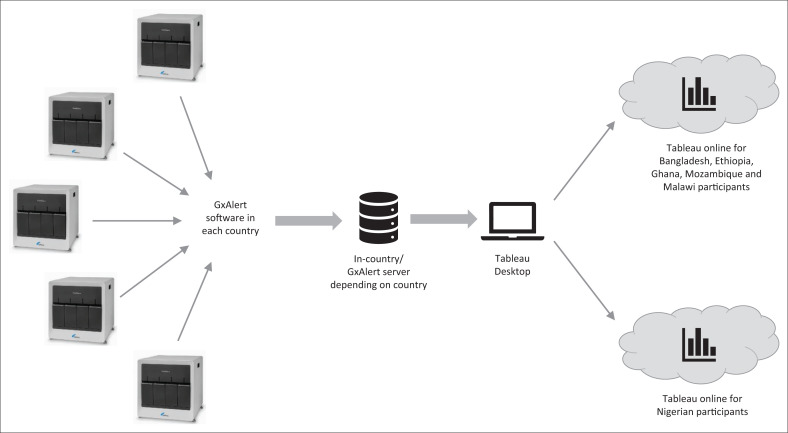
General informatics infrastructure and data flow for TB Data Fellowship programme. Live Xpert MTB/RIF data from each country is collected via GxAlert and stored on a in-country or private cloud hosted GxAlert server (depending on country preference). Each data fellow is able to download an extract of their own country data to Tableau Desktop in order to create various graph-like visualisations and basic analytics. Once analysis is complete, data fellows could choose to publish a subset of these visualisations, unlinked to the data source, via a community folder on Tableau Online, allowing them to share insights, ideas and graphics with other data fellows, the ministry of health or NTP.

The programme leveraged the existing connectivity infrastructure, GxAlert, to gain access to live Xpert MTB/RIF data. In addition, each participant was provided with a Tableau Desktop and Tableau Online licence (Tableau, Seattle, Washington, United States). Tableau is a powerful data visualisation and analytics software package that is specifically aimed at helping people understand large amounts of data through the creation of structured storyboards, dashboards and visual representations. SystemOne developed the server architecture to enable participants to extract live country data from GxAlert and to import it into Tableau (Figure 1). This allowed participants to safely interact with data, enforce the necessary patient privacy and country-specific data permissions, and generate visualisations to allow them to share this with the NTP, neighbouring disease programmes, the ministry of health or donors, via Tableau Online (Figure 1).

## Training structure

Two pilot training programmes were undertaken: the first accepted participants from five countries supported by the global Challenge TB project, funded by the United States Agency for International Development. The first countries were Bangladesh (*n* = 2), Ethiopia (*n* = 2), Mozambique (*n* = 2), Ghana (*n* = 1) and Malawi (*n* = 1). The second programme accepted participants from Nigeria only (*n* = 12). The training lasted 12 months and was structured in two parts: a 1-week in-person classroom training followed by an 11-month remote training and mentorship.

### Classroom training

The in-person classroom training consisted of a 1-week intensive centralised training, the first of which was held in Johannesburg, South Africa from 9–13 July 2018, and the second in Abuja, Nigeria from 24–28 September 2018. During the first three days of each session, participants were trained extensively on Tableau Desktop v2018.1 software (Tableau, Seattle, Washington, United States). This was followed by two days of training on how to integrate and interpret GxAlert tuberculosis data in Tableau, perform basic data analysis, and prepare visualisations to improve the interpretation and reporting of tuberculosis data.

### Remote training and mentorship

For 11 months following the classroom training, participants received monthly training and mentorship remotely via 2-hour Skype sessions as two separate groups, depending on which classroom training session they attended. SystemOne designed these sessions to allow in-depth data analysis and development of visualisations to improve understanding, with a focus on the development of data-driven recommendations for programmatic improvement. To promote a platform for sharing of data, insights and best practices, each month participants were required to complete assignments and publish their developed visualisations and insights on the Tableau online server for the entire group to see. This encouraged collaboration among members of the groups.

## Focus areas of the training

Through the 12-month programme, participants were taught how to understand GeneXpert tuberculosis diagnostic and operational data being collected by the GxAlert platform and to translate this data into insights about their respective tuberculosis programmes (Table 1). They identified programme gaps and appropriate intervention needs in different programme areas (Table 1), while learning how to lower operating costs (by reducing supervision frequency and troubleshooting services), improve the quality of the programme and manage the programme more effectively.

**TABLE 1:** Key topics covered during the 2018 TB Data Fellowship training.

Area	Data collected	Topics covered
Disease burden	MTB/RIF results Probe data Semi-quantitative values (cycle thresholds)	TB positivity and RIF positivity rates and trends Diagnostic algorithm adherence Epidemiological characteristics of circulating strains Identification of ‘hotspots’ to inform design of targeted case-finding and interventions
Patient services	Demographic data (age, sex, treatment history, etc.)	Identify which populations are underserved Identify where interventions are working Identify where interventions are needed Tailoring diagnostic and treatment strategies Improved reporting against key programme indicators
Programme monitoring	Testing numbers Instrument and module serial numbers Geographic locations of laboratories	Monitoring of test numbers and trends Growth of testing fleet and placement, gaps, absorption by the health system Instrument and module status and downtimes Instrument and module utilisation rates Monitoring progress towards testing targets
Quality monitoring	Error, invalid and no results Error codes External quality assurance and proficiency testing results	Monitoring error, invalid and no result rates and trends Reportable versus unreportable results (loss of tests) and wastage Monitoring quality of the programme Error code interpretation to target specific intervention needs, for example, re-training needs, power needs, environmental issues Identifying sites needing support and supervision Identifying specific users needing support Identify sites performing quality assurance and monitoring quality of testing
Inventory tracking	Reagent lot numbers Reagent expiry dates	Monitoring of levels of reagents and consumable stock and expiry Forecasting Prevention of stock-outs Supply chain improvement Identification of reagent lot numbers with high invalid or error rates Cartridge age and relationship to invalid or error rates

Note: Several areas of learning were covered during the 12-month programme held for Bangladesh, Ethiopia, Ghana, Mozambique, Malawi and Nigerian participants.

MTB, *Mycobacterium tuberculosis*; RIF, rifampicin; TB, tuberculosis.

## Key insights from the programme

The training has yielded new country-level insights and programme improvements due to improved data use and decision making as demonstrated by numerous technical reports, conference abstracts and presentations. For example, in Bangladesh, participants have used data about instrument utilisation rates to influence the NTP to improve referral mechanisms for underperforming sites and further optimise GeneXpert placement within their NTP to better meet testing demand.^12^ These insights have also helped the NTP plan for future placement of additional GeneXpert machines. In Ethiopia, analysis of instrument utilisation and subsequent proactive monitoring have led to an improvement in utilisation, from 28% to 75%.^13^ Such dramatic improvements can translate immediately into programme return on investment – whereby tuberculosis programmes can make existing resources go much farther than expected and deploy resources more effectively when receiving future grants or allocating domestic budgets.

By teaching participants how to monitor unreportable test rates or the number of tests resulting in errors, no results and invalid results, programme efficiency and response speed can be improved. Unreportable or unsuccessful tests do not provide a clinically valid result to the patient and thus need to be repeated. Besides the cost in ‘lost’ cartridges, when one considers that the actual cost per test performed has been estimated at $23.00 (United States dollors [USD]) and the cost per diagnosis at $99.00 USD,^14^ unsuccessful tests represent a significant cost to the health system. The majority of unsuccessful tests are due to error results and can, to a large extent, be corrected. Unfortunately, countries seldom know how to interpret error codes to inform appropriate corrective actions. The TDF helped participants categorise error codes according to their suspected sources and, through doing this, identify the most frequent types of errors to troubleshoot while pinpointing the sites needing supervision and follow-up. This real-time support is less costly compared to conventional monitoring, which requires a person to visit sites to troubleshoot issues, without any understanding of which issues pertain to which sites. Across all participating countries, the majority of errors were user or technical errors. These errors are associated with incorrect specimen processing or volumes added to the cartridge.^15^ For example, in Ghana, up to 67% of error results were identified as user related, and this insight has led to the introduction of refresher training and targeted supervision for sites.^16^ The same issue was identified in Nigeria and Bangladesh, where both programmes have managed to reduce their national error rates due to targeted supervision, refresher training for laboratory staff and regular feedback to laboratories aimed at addressing the high incidence of these user related errors.^12,17^

Another focus area of the TDF training that has led to significant programmatic improvement is the monitoring of testing fleet and instrument downtime. A challenge faced by many tuberculosis programmes is that GeneXpert instruments are often located at remote facilities, leading to delays in maintenance and replacement of broken modules. By monitoring trends in how instruments report in real-time through connectivity tools, the Bangladesh NTP are now identifying directly when modules are down or instruments require calibration. Through this real-time monitoring of instrument performance, Bangladesh has managed to reduce instrument maintenance turn-around time from anywhere between 5 and 14 months to just 2 weeks, and is now also maintaining 90% module functionality.^12^

### Ethical considerations

Ethical clearance was not required for this study.

## Discussion

Various connectivity solutions exist to collect diagnostic data, some of which have already been adopted widely. Connectivity tools can play a major role in addressing many of the challenges that tuberculosis programmes face by facilitating the central collection and aggregation of diagnostic instrument data so that it can be analysed. However, the introduction of connectivity tools is not sufficient to ensure improved programme management. Well-functioning health systems need to utilise this data at all levels in order to drive evidence-based decisions and interventions to improve the quality of care provided.^18^

To our knowledge, the TDF programme is the first of its kind to build capacity and resources in low- and middle-income countries for the analysis of tuberculosis data collected from connected diagnostics. To address sustainability, the programme provided participants with the much-needed tools required to drive data analytics, counting on participants to lead the ongoing analysis of tuberculosis-related health data from the national GeneXpert programme in their respective countries. We chose Tableau software as a data analytics and visualisation tool, because it enables users to explore, manipulate and create visual representations of large amounts of data in order to produce insights as well as to communicate those insights to a broader audience. We leveraged an existing connectivity footprint, namely the GxAlert system (SystemOne, LLC, Northampton, Massachusetts, United States), to gain access to GeneXpert tuberculosis data within each respective country, but the programme is translatable to any connectivity platform collecting tuberculosis GeneXpert data. While the initial pilot programme focused on tuberculosis, the intent is to expand into related disease streams and diagnostics within the ministry of health, such as HIV. By providing an online tool (Tableau Online) where participants could post their visualisations and insights, the programme also provides a platform whereby countries can share best practices and help to create value.

The TDF programme has already seen rapid development and analysis of country key performance indicators leading to immediate publications, programme engagements and strengthening.^12,13,16,17^ By using the data to recognise programme gaps and identify needs, issues and priorities, participants have been equipped to help develop their national strategies, address challenges and inform data-driven decision making.

### Conclusion

The programme empowers local ministry of health, NTP and national tuberculosis reference laboratory staff to lead the analysis of tuberculosis-related data, discover and fix critical inefficiencies, and provide high-level technical and operational support to tuberculosis programmes. Through data-driven, actionable recommendations, the TDF helps to strengthen, improve and complement NTPs in low- and middle-income countries and, ultimately, improve healthcare delivery.

Lessons learned

- Delivery of test data in a timely manner through connectivity platforms is critical to successful reporting and monitoring of tuberculosis programmes.
- Countries lack the capacity and experience in interpretation of diagnostic instrument data to be able to translate it into targeted interventions to improve programme performance.
- Selection of participants for this type of training needs to be more targeted to specific positions within the ministry of health and NTP programme to ensure better skills transfer.
- There was a decline in participant engagement toward the end of the 12-month training programme. In future, training should be accelerated towards a 3–6 month programme in order to avoid any decline.
- There is a need for institutionalisation of this kind of training by the World Health Organization, ministry of health, donors, implementers and partners, as connectivity becomes one of the cornerstone tools in data capture and management.
